# Snaring of the right ventricular lead during cavotricuspid isthmus ablation

**DOI:** 10.1002/ccr3.813

**Published:** 2017-02-05

**Authors:** Yousef H. Darrat, Morales X. Gustavo, Cristen Kelly Waespe, John C. Gurley, Claude S. Elayi

**Affiliations:** ^1^ Gill Heart Institute and Veterans Administration Medical Center, Division of CardiologyUniversity of KentuckyLexingtonKentuckyUSA

**Keywords:** Ablation, atrial flutter, implantable defibrillator, transvenous lead

## Abstract

The presence of a right ventricular (RV) lead may interfere with cavotricuspid isthmus (CTI) ablation. We present a new option of lifting the RV lead from the CTI allowing a successful ablation of a CTI‐dependent flutter without compromising lead integrity and functionality.

## Introduction

Cavotricuspid isthmus (CTI) ablation is a standard therapy for typical atrial flutter. Many patients requiring CTI ablation have a cardiac implantable electronic device (CIED). The right ventricular (RV) lead crosses the tricuspid valve and is typically in close proximity to or in direct contact with the CTI, thus may interfere with radiofrequency (RF) ablation.

## Case Presentation

**Figure 1:**
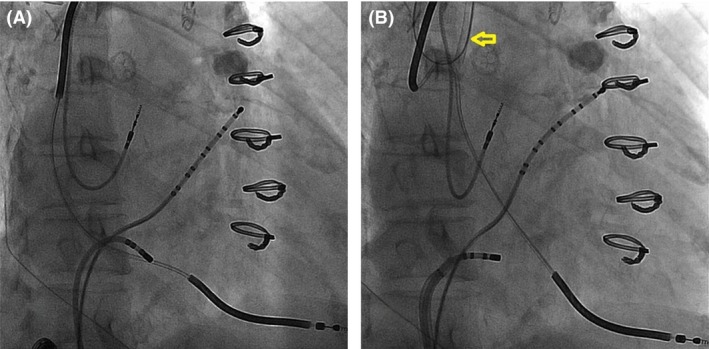
(A) RV lead on CTI in right anterior oblique view. (B) RV lead lifted using snare, indicated by the arrow, in the same right anterior oblique view. CTI, Cavotricuspid isthmus; RV, right ventricular.

**Figure 2:**
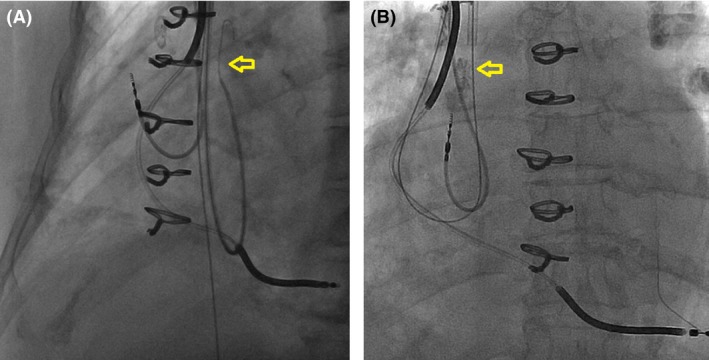
(A) This image shows the headhunter catheter around the ICD lead that allowed the advancement of the 0.035‐inch guide wire around the lead body (arrow). (B) The catheter was removed, before the Amplatz GooseNeck snare “grabbed” and secured the tip of the guide wire with removal of the catheter.

A 67‐year‐old female with ischemic cardiomyopathy was followed at an outside hospital for recurrent defibrillator shocks. The patient had inappropriate therapy due to recurrent atrial flutter with rapid ventricular response uncontrolled with medical therapy. CTI ablation was attempted but was not completed due to the fear of damaging the RV lead that was in the path of ablation. The patient was subsequently referred to our institution. We offered repeat CTI ablation, but the patient was initially reluctant because of the potential interaction between the RV lead and ablation catheter. Therefore, we proposed an alternative to avoid delivering RF energy in close proximity to the lead. A decapolar (Bard Electrophysiology, Lowell, MA) catheter was placed in coronary sinus, and an irrigated 3.5‐mm tip F‐curve ablation catheter (ThermoCool Biosense‐Webster, Diamond Bar, CA) was positioned across the CTI. The RV lead was an eight‐year‐old Medtronic Sprint Quattro Secure Model 6947. However, the lead was located in the preferential target site of CTI ablation, representing an obstacle for adequate contact of the ablation catheter with the endocardium (Fig. 1A). Ablation was therefore initially performed laterally and septally to avoid direct contact with the RV lead, but conduction block across the CTI could not be achieved. At this point, the decision was made to temporarily move the RV lead body to provide sufficient access to the CTI. A 6 French H1 “Headhunter” catheter (Cook Medical, Bloomington, IN) was inserted via right internal jugular vein access and used to place a 0.035‐inch guide wire around the lead body, and then, an Amplatz GooseNeck snare (ev3 Inc., Plymouth, MN) was used to secure the distal tip of the guide wire (Fig. 2). Traction to the guide wire ends gently lifted the lead from its original position at the CTI (Fig. 1B). With a “lead‐free” CTI, ablation was performed until bidirectional conduction block was achieved. After ablation, the 0.035‐inch wire was removed, returning the RV lead to its original position. Immediate and subsequent device interrogations revealed no significant changes in the lead parameters up to one‐year follow‐up.

## Discussion

The presence of an RV lead on the CTI may represent an obstacle in the path of CTI ablation. Furthermore, potential interactions between RF and CIEDs have been previously reported 1, 2, 3, 4. There is general consensus among Heart Rhythm Society and American Society of Anesthesiologists members that the operator performing RF ablation should avoid direct contact between the ablation catheter and lead system 5. CIED manufacturers have similar recommendation regarding this issue (Medtronic. CRDM Technical Services Standard Letter 2013, St. Jude Medical: Effect of RF ablation on St. Jude Medical Implantable Cardiac Rhythm Devices 2009). The technique described here provides an option to allow CTI ablation while temporarily removing the RV lead body from the CTI. The procedure of snaring the RV is feasible and relatively simple. It can be an attractive option in certain cases where there could be interference between the ablation catheter and the transvenous lead.

## Conclusion

The presence of an RV lead may potentially interfere with CTI‐dependent atrial flutter ablation. We describe a technique to temporarily displace the lead from the CTI to allow a “lead‐free” successful ablation.

## Authorship

YHD: design of the work and drafting the article. GXM: critical revision of the article. CKW: critical revision of the article. JCG: critical revision of article and final approval. CSE: conception of the work, critical revision of the article and final approval.

## Conflict of Interest

None declared.

## Supporting information


**Video S1.** The video demonstrates “snaring” the right ventricular lead and lifting it away in order to facilitate Cavotricuspid Isthmus catheter ablation.
